# Supplementary material for: MetaRibo-Seq measures translation in microbiomes
Source: Nat Commun. 2020 Jun 29;11:3268. doi: 10.1038/s41467-020-17081-z (PMC7324362; doi:10.1038/s41467-020-17081-z)
Supplement: Supplementary file 10 — Supplementary Data 7 [file 41467_2020_17081_MOESM10_ESM.zip › File2/Confidence_High_Taxonomy/85008_out.krona.html]

Javascript must be enabled to view this page.

members
magnitude
magnitudeUnassigned
count
unassigned
taxon
rank

85008\_out

9

superkingdom
2
9

phylum
976
7

200643
7
class

7
171549
order

family
171552

SRS013800\_contig\_number\_21915
6
1

838
genus
1
5

SRS024435\_contig\_number\_34423

1280674
2
species

SRS104912\_contig\_number\_1239SRS149325\_contig\_number\_11966


SRS049959\_contig\_number\_13091
species
1262925
1

159272
1

SRS049446\_contig\_number\_3732
species

family
815
1

genus
816
1

46506
1

SRS098073\_contig\_number\_13284
species

1224
1
phylum

species

SRS144537\_contig\_number\_47123
1262987
1

phylum
1
1239

1
186801
class

1
186802
order

family
216572
1

genus
1
459786

1
876091
species

SRS019808\_contig\_number\_contig-100\_1511.1512
